# The intervention seasons of thoracic endovascular aortic repair impacted the outcomes for patients with type B aortic dissection

**DOI:** 10.3389/fcvm.2023.1100075

**Published:** 2023-03-21

**Authors:** Kaiwen Zhao, Hongqiao Zhu, Xiaomin He, Taiping Liang, Yudong Sun, Jian Zhou, Zaiping Jing

**Affiliations:** ^1^ Department of Vascular Surgery, The First Affiliated Hospital of the Navy Medical University, Shanghai, China; ^2^ Depaertment of General Surgery, Jinling Hospital, Medical School of Nanjing University, Nanjing, China

**Keywords:** aortic dissection, stent graft, seasonal, adverse events, outcomes

## Abstract

**Purpose:**

The objective of this research was to investigate whether seasonal variations influence the outcomes of type B aortic dissection (TBAD) patients with thoracic endovascular aortic repair (TEVAR).

**Patients and methods:**

From 2003 to 2020, a retrospective cohort study was performed, which included 1,123 TBAD patients who received TEVAR. Medical records were used to gather data on baseline characteristics. Outcomes including all-cause mortality and aortic-related adverse events (ARAEs) were tracked and analyzed.

**Results:**

Of the 1,123 TBAD patients in this study, 308 received TEVAR in spring (27.4%), 240 cases in summer (21.4%), 260 cases in autumn (23.2%), and 315 cases in winter (28.0%). Patients in the autumn group had a significantly lower risk of 1-year mortality than those in the spring group (hazard ratio: 2.66, 95% confidence interval: 1.06–6.67, *p* = 0.037). Kaplan–Meier curves revealed that patients who underwent TEVAR in autumn had a lower risk of 30-day ARAEs (*p* = 0.049) and 1-year mortality (*p* = 0.03) than those in spring.

**Conclusion:**

This study confirmed that TEVAR operated in autumn for TBAD was associated with a lower risk of 30-day ARAEs and 1-year mortality than in spring.

## Introduction

Type B aortic dissection (TBAD) is a life-threatening state and is classified as involving the aorta distal to the subclavian artery (1). Thoracic endovascular aortic repair (TEVAR) has been proven to be effective in improving the outcomes and producing favorable aortic remodeling in TBAD patients (2–4). However, TEVAR still has several flaws, with a number of postoperative adverse events undermining its advantages, such as retrograde type A dissection (RTAD), spinal cord ischemia, and endoleak (5). As a consequence, identifying risk factors that influence outcomes of TBAD patients is critical for the reasonable application of TEVAR.

For the past two decades, the impacts of seasonal variations on cardiovascular disorders have been gaining special attention (6). The incidence of acute aortic dissection (AD) was found to be chronobiological, with the highest in winter and the lowest in summer (7). Low ambient temperatures and temperature differences between days have been linked to an increased risk of acute AD onset (8). Seasonal fluctuations in atmospheric pressure also contributed to a higher risk of aortic aneurysm rupture (9). In this context, seasonal variations may also impact the aortic remodeling and outcomes of AD patients after surgery. A few previous studies have indicated that the start of symptoms and the season of admission raise the risk of in-hospital mortality in patients with type A aortic dissection (TAAD) (10, 11). However, it remains unclear whether the chronobiology of TEVAR intervention affects the prognosis of TBAD patients.

The purpose of this retrospective research was to examine the outcomes of TEVAR for TBAD patients in various seasons.

## Materials and methods

Between February 2003 and December 2020, 1,123 consecutive TBAD patients who underwent TEVAR were reviewed at the First Affiliated Hospital of the Navy Medical University (Changhai Hospital), Shanghai, China. A multidisciplinary board comprising vascular surgeons, cardiologists, and anesthetists reviewed all the cases. To determine the final survival status, hospital admission records were reviewed or phone calls were made.

Inclusion criteria for this study were as follows: (1) age 18–80 years, (2) pathology of TBAD (as determined by Society for Vascular Surgery (SVS's) 15-standard anatomic categorization of entry tear ≥1) (12), and (3) TEVAR performed between February 2003 and December 2020.

Patients were excluded if they (1) had Marfan's syndrome, Turner's syndrome, bicuspid aortic valve, Bechet's disease, Ehlers–Danlos syndrome, giant cell arteritis, ankylosing spondylitis, or Takayasu’s arteritis; (2) refused intervention and received medical treatment in outpatient; or (3) had AD caused by trauma, iatrogenic injury, or intramural hematoma.

### Study protocol

Information regarding dates of symptom onset, TEVAR intervention, and clinical outcomes was obtained from the history of the present illness in the patient’s medical record. For the purpose of analysis, patients were categorized into four groups based on the seasons of TEVAR intervention: spring (March, April, and May), summer (June, July, and August), autumn (September, October, and November), and winter (December, January, and February).

### Definition

Aortic-related adverse events (ARAEs) included rupture, endoleak (types I and III), retrograde type A dissection, aortic dilatation, and malperfusion after TEVAR (13).

### Statistical analysis

For values that were categorical variables, the data were presented as *n* (%), and for those that were continuous variables, the data were presented as mean ± SD. *χ*^2^ or Fisher exact test were used to compare categorical variables, whereas the Student *t*-test was used to compare continuous variables.

Overall survival and freedom from ARAEs were estimated using a Kaplan–Meier analysis, with log-rank tests used to distinguish between the Kaplan–Meier curves. In univariate analysis, Cox analysis was performed to investigate the influence of each significant variable on the incidence of all-cause mortality and ARAEs. In addition, the nonlinear association between TEVAR intervention dates and log relative risk (logRR) of mortality and ARAEs was investigated *via* restricted cubic spline analysis. Statistical significance was defined as a *p*-value of less than 0.05. The data were analyzed using the statistical tool R version 3.6.3 (R Project, Vienna, Austria).

## Results

### Baseline characteristics

**Figure 1:**
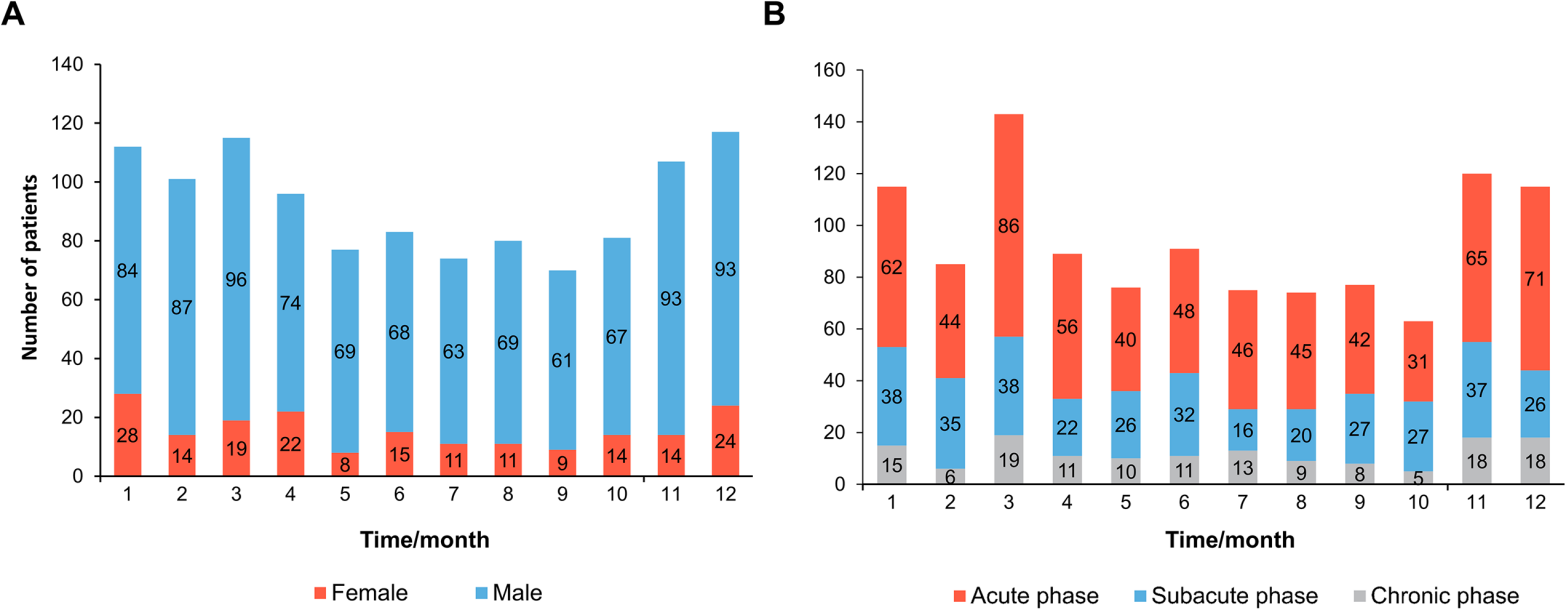
The frequency distribution histogram for TBAD patients receiving TEVAR varied by month. (**A**) Population distribution according to the time of TBAD onset. (**B**) Population distribution according to the time of TEVAR intervention. TBAD, type B aortic dissection; TEVAR, thoracic endovascular aortic repair.

A total of 1,123 patients were enrolled in this study, with 308 patients undergoing TEVAR in spring, 240 in summer, 260 in autumn, and 315 in winter. Table 1 demonstrates the baseline characteristics of the study population stratified by the seasons of TEVAR. The demographic distribution of symptom onset months is shown in Figure 1A. The demographic distribution of TEVAR intervention months is shown in Figure 1B.

**Table 1:** Baseline characteristics of type B aortic dissection patients stratified with seasons of thoracic endovascular aortic repair.

Variables	Spring (*n* = 308)	Summer (*n* = 240)	Autumn (*n* = 260)	Winter (*n* = 315)	*p*-value
Age, years	58.2 ± 13.3	59.8 ± 12.8	57.5 ± 12.5	59.0 ± 13.4	0.205
Male	251 (81.5%)	202 (84.2%)	223 (85.8%)	257 (81.6%)	0.459
BMI (kg/m^2^)	24.6 ± 3.6	24.5 ± 3.9	24.0 ± 3.5	24.6 ± 3.3	0.318
Smoking	164 (53.2%)	134 (55.8%)	135 (51.9%)	153 (48.6%)	0.380
Drinking	51 (16.6%)	51 (21.2%)	44 (16.9%)	52 (16.5%)	0.428
SBP at admission (mmHg)	136.0 ± 22.0	137.6 ± 19.8	137.5 ± 19.1	138.4 ± 22.7	0.552
DBP at admission (mmHg)	82.4 ± 12.7	81.9 ± 10.6	82.2 ± 10.2	82.2 ± 11.3	0.975
Hypertension	243 (78.9%)	178 (74.2%)	192 (73.8%)	231 (73.3%)	0.357
Diabetes mellitus	15 (4.9%)	28 (11.7%)	24 (9.2%)	26 (8.3%)	0.034
Stroke	14 (4.5%)	15 (6.2%)	18 (6.9%)	18 (5.7%)	0.663
CAD	10 (3.8%)	18 (5.8%)	17 (7.1%)	19 (6.0%)	0.459
COPD	32 (10.4%)	33 (13.8%)	22 (8.5%)	36 (11.4%)	0.289
CKD	16 (5.2%)	16 (6.7%)	18 (6.9%)	14 (4.4%)	0.53
WBC count (×10^9^/L)	8.7 ± 3.5	8.4 ± 3.6	8.6 ± 3.7	9.2 ± 3.7	0.1
Platelet count (×10^9^/L)	209.6 ± 91.5	196.0 ± 74.2	210.5 ± 87.0	210.8 ± 84.2	0.181
Creatinine (µmol/L)	103.1 ± 114.4	103.6 ± 106.0	107.7 ± 116.3	109.1 ± 132.4	0.921
FDP (μg/mL)	13.6 ± 17.9	13.6 ± 19.9	13.8 ± 17.9	15.4 ± 22.7	0.742
**CTA parameters**
Arch type					0.381
I	82 (26.6%)	64 (26.7%)	44 (16.5%)	100 (31.7%)	
II	56 (18.2%)	48 (20.0%)	35 (13.5%)	54 (17.1%)	
III	170 (55.2%)	128 (53.3%)	181 (69.6%)	161 (51.2%)	
Diameters of maximum descending aorta (mm)	43.7 ± 17.6	43.2 ± 11.2	40.9 ± 9.8	41.6 ± 11.5	0.458
Length of aortic dissection (mm)	447.9 ± 112.3	405.4 ± 150.9	420.3 ± 150.6	362.7 ± 139.7	0.070
Proximal thrombosis of false lumen					0.255
Patent	129 (42.0%)	103 (42.9%)	99 (38.0%)	122 (38.7%)	
Partial	100 (32.4%)	79 (32.9%)	105 (40.6%)	94 (29.8%)	
Complete	48 (15.5%)	33 (13.8%)	39 (15.0%)	55 (17.4%)	
ULP	31 (10.0%)	25 (10.4%)	17 (6.5%)	44 (14.0%)	
Echocardiography parameters
LVEF (%)	61.6 ± 5.2	62.6 ± 5.7	61.8 ± 4.6	61.4 ± 5.2	0.182
Pericardial effusion	18 (5.8%)	17 (7.1%)	10 (3.8%)	28 (8.9%)	0.097
Pleural effusion	100 (32.5%)	71 (29.6%)	78 (30.0%)	109 (34.6%)	0.546
Malperfusion
Superior mesenteric arteries	2 (0.6%)	0 (0.0%)	0 (0.0%)	2 (0.6%)	0.359
Renal arteries	6 (1.9%)	6 (2.5%)	11 (4.2%)	16 (5.1%)	0.128
Common hepatic arteries	0 (0.0%)	0 (0.0%)	0 (0.0%)	1 (0.3%)	0.463
Lower extremity arteries	3 (1.0%)	6 (2.5%)	3 (1.2%)	2 (0.6%)	0.238

BMI, body mass index; SBP, systolic blood pressure; DBP, diastolic blood pressure; CAD, coronary artery disease; COPD, chronic obstructive pulmonary disease; CKD, chronic kidney disease; FDP, fibrin degradation product; CTA, computed tomographic angiogram; LVEF, left ventricular ejection fraction; ULP, ulcer-like projection.

Values are *n* (%), mean ± SD, or median (25th–75th percentile). Categorical variables were presented as *n* (%)

The mean ages of the four groups were 58.2 ± 13.3 years (spring), 59.8 ± 12.8 years (summer), 57.5 ± 12.5 years (autumn), and 59.0 ± 13.4 years (winter) (*p* = 0.205). Male prevalence was not significantly different between the four groups (spring vs. summer vs. autumn vs. winter, 81.5% vs. 84.2% vs. 85.8% vs. 81.6%, *p* = 0.459). The difference in body mass index, smoking, and drinking between the four groups was rather negligible (*p* = 0.318, 0.380, and 0.428, respectively). No significant difference can be spotted in the admission systolic blood pressure and diastolic blood pressure between the four groups (*p* = 0.552 and 0.975, respectively). The differences in the incidences of hypertension, previous stroke, chronic obstructive pulmonary disease (COPD), and chronic kidney disease (CKD) between the four groups were also insignificant (all *p* > 0.05). However, the incidence of diabetes in the spring group was significantly lower than that in the other group (4.9% vs. 11.7% vs. 9.2% vs. 8.3%, *p* = 0.034). No difference was observed in the hematological indices, such as the white blood cell count, platelet count, creatine, and fibrin degradation product in the four groups (all *p* > 0.05). The imaging results showed that arch type, diameters of the maximum descending aorta, length of the AD, proximal thrombosis of the false lumen, and malperfusion have no statistical differences (all *p* > 0.05). In addition, there was no difference in the echocardiography parameters including left ventricular ejection fraction (LVEF), pericardial effusion, and pleural effusion presenting symptoms in the four groups (all *p* > 0.05).

### Endovascular procedure and antihypertensive drugs

In the present study, there were no differences between the four groups in the acute, subacute, and chronic phases (*p* = 0.649). No difference was found in the mode of surgery, which consisted of the chimney technique, adjunctive procedures, and hybrid approach (all *p* > 0.05). Angiotensin-converting enzyme inhibitor (ACEI), angiotensin receptor blocker (ARB), β-blocker, and calcium channel blocker (CCB) were the most commonly prescribed medications at discharge, with no significant difference between the four groups (all *p* > 0.05). Patients in the spring group were found to have less diuretic than those in the other three groups (*p* = 0.039) (Table 2).

**Table 2:** Endovascular procedure and antihypertensive drugs stratified with seasons of thoracic endovascular aortic repair.

Variables	Spring (*n* = 308)	Summer (*n* = 240)	Autumn (*n* = 260)	Winter (*n* = 315)	*p*-value
Hospital stays of post-TEVAR (days)	12.1 ± 7.2	12.4 ± 6.9	13.5 ± 7.4	12.4 ± 7.2	0.105
Timing of operation					0.649
Acute phase	182 (59.1%)	139 (57.9%)	138 (53.1%)	177 (56.2%)	
Subacute phase	86 (27.9%)	68 (28.3%)	91 (35.0%)	99 (31.4%)	
Chronic phase	40 (13.0%)	33 (13.8%)	31 (11.9%)	39 (12.4%)	
General anesthesia	242 (78.6%)	189 (78.8%)	206 (79.2%)	249 (79.0%)	0.998
Chimney technique	57 (18.5%)	50 (20.8%)	48 (18.5%)	44 (14.0%)	0.182
Adjunctive procedure	59 (19.2%)	44 (18.3%)	51 (19.6%)	59 (18.7%)	0.985
Hybrid approach	5 (1.6%)	3 (1.2%)	5 (1.9%)	3 (1.0%)	0.777
Medications at discharge
ACEI	19 (6.2%)	22 (9.2%)	18 (6.9%)	19 (6.0%)	0.471
ARB	81 (26.3%)	64 (26.7%)	82 (31.5%)	92 (29.2%)	0.497
β-blocker	145 (47.1%)	102 (42.5%)	118 (45.4%)	153 (48.6%)	0.532
CCB	161 (52.3%)	128 (53.3%)	151 (58.1%)	182 (57.8%)	0.375
Diuretic	43 (14.0%)	40 (16.7%)	56 (21.5%)	68 (21.6%)	0.039

ACEI, angiotensin-converting enzyme inhibitor; ARB, angiotensin receptor blocker; CCB, calcium channel blocker.

Values are *n* (%) or mean ± SD.

### Thirty-day and 1-year follow-up outcomes

The mean hospital stay after TEVAR of the four groups was 12.1 ± 7.2 days (spring), 12.4 ± 6.9 days (summer), 13.5 ± 7.4 days (autumn), and 12.4 ± 7.2 days (winter) (*p* = 0.105) (Table 2). The mean follow-up time of the four groups was 37.3 ± 45.3 months (spring), 40.0 ± 43.3 months (summer), 35.4 ± 38.8 months (autumn), and 35.2 ± 35.1 months (winter) (*p* = 0.817). The details of 1-year ARAEs of TBAD patients are shown in Table 3. Cox analysis revealed that there was no significant difference between the four groups in 1-year ARAEs [hazard ratio (HR): 1.55, 95% CI: 0.86–2.79; *p* = 0.149]. It demonstrated that the risk of 1-year mortality was higher in the spring group than that in the autumn group (HR: 2.66, 95% CI: 1.06–6.67; *p* = 0.036). In contrast, the risk of a 1-year endoleak was the opposite, which was lower in the spring (HR: 0.21, 95% CI: 0.04–0.98; *p* = 0.047) (Table 4). In addition, no differences in the outcomes were found between the four groups divided according to the symptom onset seasons (all *p* > 0.05) (Supplementary Table S1).

**Table 3:** Details of 1-year ARAEs stratified with seasons of TEVAR.

Variables	Spring (*n* = 308)	Summer (*n* = 240)	Autumn (*n* = 260)	Winter (*n* = 315)	*p*-value
Cumulative incidence of all-cause death	7.18% (3.99–10.26)	4.41% (1.52–7.2)	2.76% (0.54–4.92)	5.15% (2.46–7.76)	0.161
Cumulative incidence of ARAEs	11.67% (7.68–15.48)	10.32% (5.88–14.55)	8.02% (4.28–11.62)	13.4% (9.13–17.47)	0.254
Cumulative incidence of type I/III endoleak	3.65% (1.39–5.86)	1.05% (0–2.5)	1.38% (0–2.92)	2.75% (0.72–4.74)	0.157
Cumulative incidence of aortic dilation	1.92% (0.23–3.58)	3.15% (0.62–5.62)	0.88% (0–2.10)	2.41% (0.48–4.30)	0.505
Cumulative incidence of RTAD	3.65% (1.39–5.86)	1.05% (0–2.5)	1.38% (0–2.92)	2.75% (0.72–4.74)	0.147
Cumulative incidence of rupture	4.52% (1.97–7.01)	0	2.24% (0.27–4.18)	3.02% (0.91–5.09)	0.023
Cumulative incidence of malperfusion	0.93% (0–2.21)	2.43% (0.29–4.52)	0	2.27% (0.45–4.07)	0.075

ARAEs, aortic-related adverse events; TEVAR, thoracic endovascular aortic repair; RTAD, retrograde type A dissection.

**Table 4:** Cox analyses of 1-year mortality and ARAEs stratified with seasons of TEVAR.

Variables	Autumn	Spring		Summer		Winter	
		HR (95% CI)	*p*-value	HR (95% CI)	*p*-value	HR (95% CI)	*p*-value
All-cause death	Reference	2.66 (1.06–6.67)	0.036	1.59 (0.57–4.47)	0.379	1.95 (0.75–5.07)	0.172
ARAEs	Reference	1.55 (0.86–2.79)	0.149	1.26 (0.66–2.40)	0.490	1.73 (0.97–3.09)	0.062
Type I/III endoleak	Reference	0.21 (0.04–0.98)	0.047	0.40 (0.10–1.49)	0.170	0.63 (0.22–1.80)	0.384
Aortic dilation	Reference	4.26 (0.50–37.44)	0.186	5.41 (0.63,46.37)	0.123	5.10 (0.61–42.38)	0.131
RTAD	Reference	2.83 (0.78–10.29)	0.114	0.70 (0.12–4.19)	0.696	1.95 (0.51–7.56)	0.331
Rupture	Reference	2.02 (0.71–5.73)	0.187	NA	0.996	1.34 (0.44–4.09)	0.611
Malperfusion	Reference	2.36 (0.85–6.4)	0.100	1.06 (0.31–3.66)	0.927	2.17 (0.77–6.09)	0.141

ARAEs, aortic-related adverse events; TEVAR, thoracic endovascular aortic repair; HR, hazard ratio; CI, confidence interval; RTAD, retrograde type A dissection.

**Figure 2:**
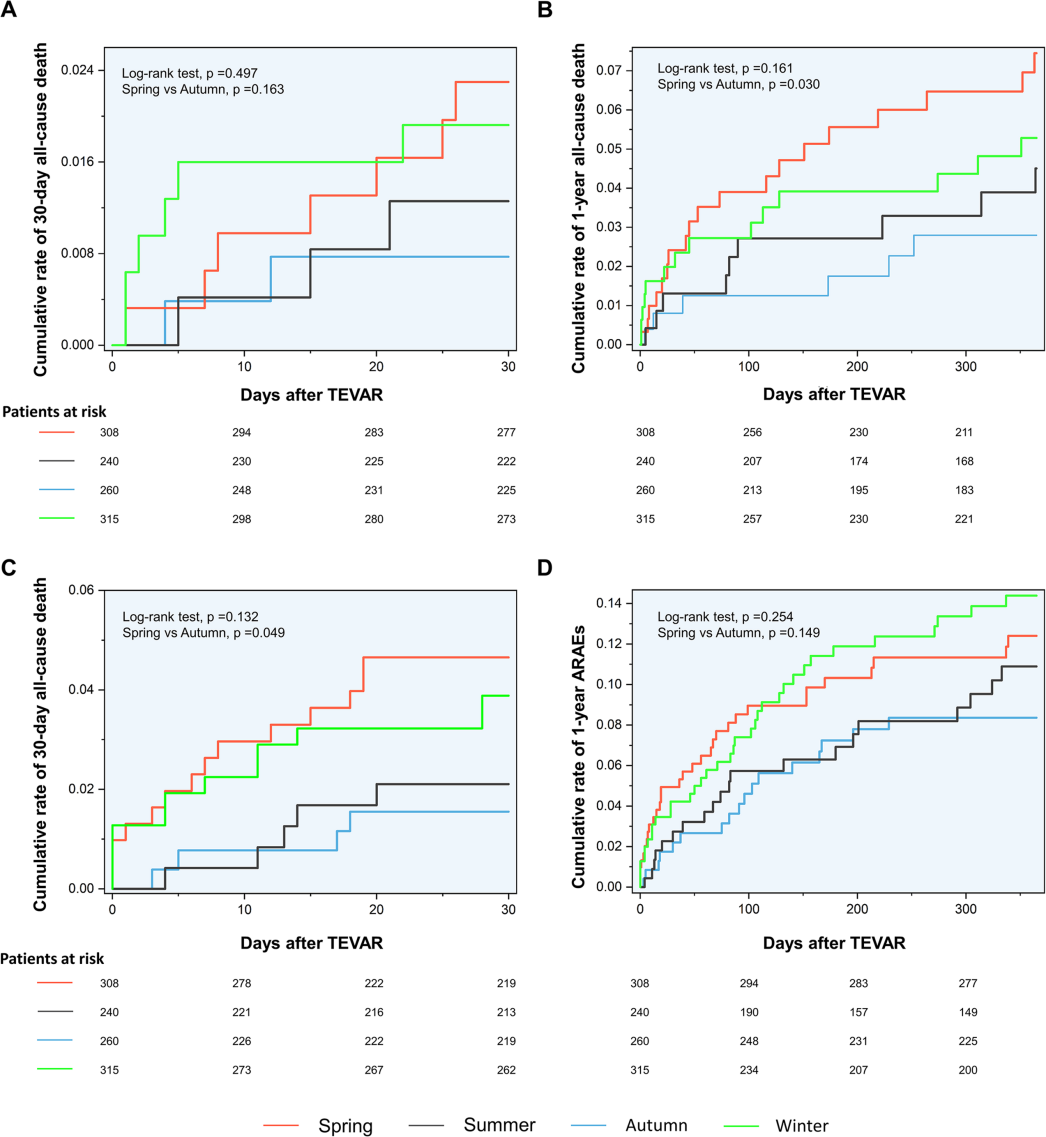
Associations of interventional dates with outcomes of TBAD patients. (**A**) Fitted curve of the data for logRRs for 30-day mortality against dates for TEVAR. (**B**) Fitted curve of the data for logRRs for 1-year mortality against dates for TEVAR. (**C**) Fitted curve of the data for logRRs for 30-day ARAEs against dates for TEVAR. (**D**) Fitted curve of the data for logRRs for 1-year ARAEs against dates for TEVAR. TBAD, type B aortic dissection; logRRs, log relative risks; ARAEs, aortic-related adverse events; CI, confidence interval.

The Kaplan–Meier curves between the spring and autumn groups are shown in Figure 2. Log-rank tests revealed a significantly increased rate of 30-day ARAEs (log-rank tests, *p* = 0.049) and 1-year all-cause deaths (log-rank tests, *p* = 0.030) in the spring group, compared with the autumn group.

In the univariate Cox regression model, the association between clinical variables and 1-year all-cause mortality was analyzed. After adjustment for confounding factors, TEVAR in the spring group was confirmed to be associated with the higher 1-year mortality than in the autumn group (HR: 2.99, 95% CI: 1.19–7.05; *p* = 0.020). There was no significant difference in 1-year mortality between the groups divided in terms of symptom onset seasons (Table 5).

**Table 5:** Univariate and multivariate cox regression analysis of 1-year all-cause mortality of type B aortic dissection patients.

Variables	Univariate analysis		Multivariate analysis	
	HR (95% CI)	*p*-value	HR (95% CI)	*p*-value
**Intervention season**
Autumn	1		1	
Spring	2.66 (1.06–6.67)	0.037	2.99 (1.19–7.50)	0.020
Summer	1.59 (0.57–4.47)	0.379	1.61 (0.57–4.53)	0.367
Winter	1.95 (0.75–5.07)	0.172	2.19 (0.84–5.72)	0.109
**Onset season**
Autumn	1			
Spring	1.88 (0.76–4.60)	0.169		
Summer	1.52 (0.58–4.00)	0.393		
Winter	1.68 (0.68–4.11)	0.259		
Age	1.02 (1.00–1.04)	0.112		
Male	1.19 (0.53–2.65)	0.671		
Smoking	0.93 (0.53–1.64)	0.810		
Drinking	0.69 (0.31–1.54)	0.365		
BMI	0.94 (0.85–1.04)	0.242		
Hypertension	1.91 (0.86–4.25)	0.114		
CAD	1.82 (0.72–4.60)	0.204		
Diabetes mellitus	1.86 (0.83–4.15)	0.129		
Stroke	3.99 (1.93–8.24)	<0.001	4.10 (1.98–8.51)	<0.001
COPD	0.74 (0.26–2.05)	0.557		
CKD	2.86 (1.28–6.37)	0.010	2.81 (1.25–6.30)	0.012
**Timing of operation**
Acute phase	1			
Subacute phase	0.96 (0.51–1.81)	0.909		
Chronic phase	0.82 (0.34–1.99)	0.666		
ACEI	0.29 (0.04–2.08)	0.217		
ARB	0.65 (0.33–1.31)	0.234		
β-blocker	0.56 (0.31–1.02)	0.060		
CCB	0.56 (0.32–0.99)	0.047		
Diuretic	0.61 (0.26–1.44)	0.261		
Chimney technique	1.07 (0.52–2.20)	0.864		
Adjunctive procedure	1.09 (0.54–2.19)	0.803		
Hybrid approach	1.50 (0.21–10.86)	0.689		

HR, hazard ratio; CI, confidence interval; BMI, body mass index; CAD, coronary artery disease; COPD, chronic obstructive pulmonary disease; CKD, chronic kidney disease; ACEI, angiotensin-converting enzyme inhibitor; ARB, angiotensin receptor blocker; CCB, calcium channel blocker.

**Figure 3:**
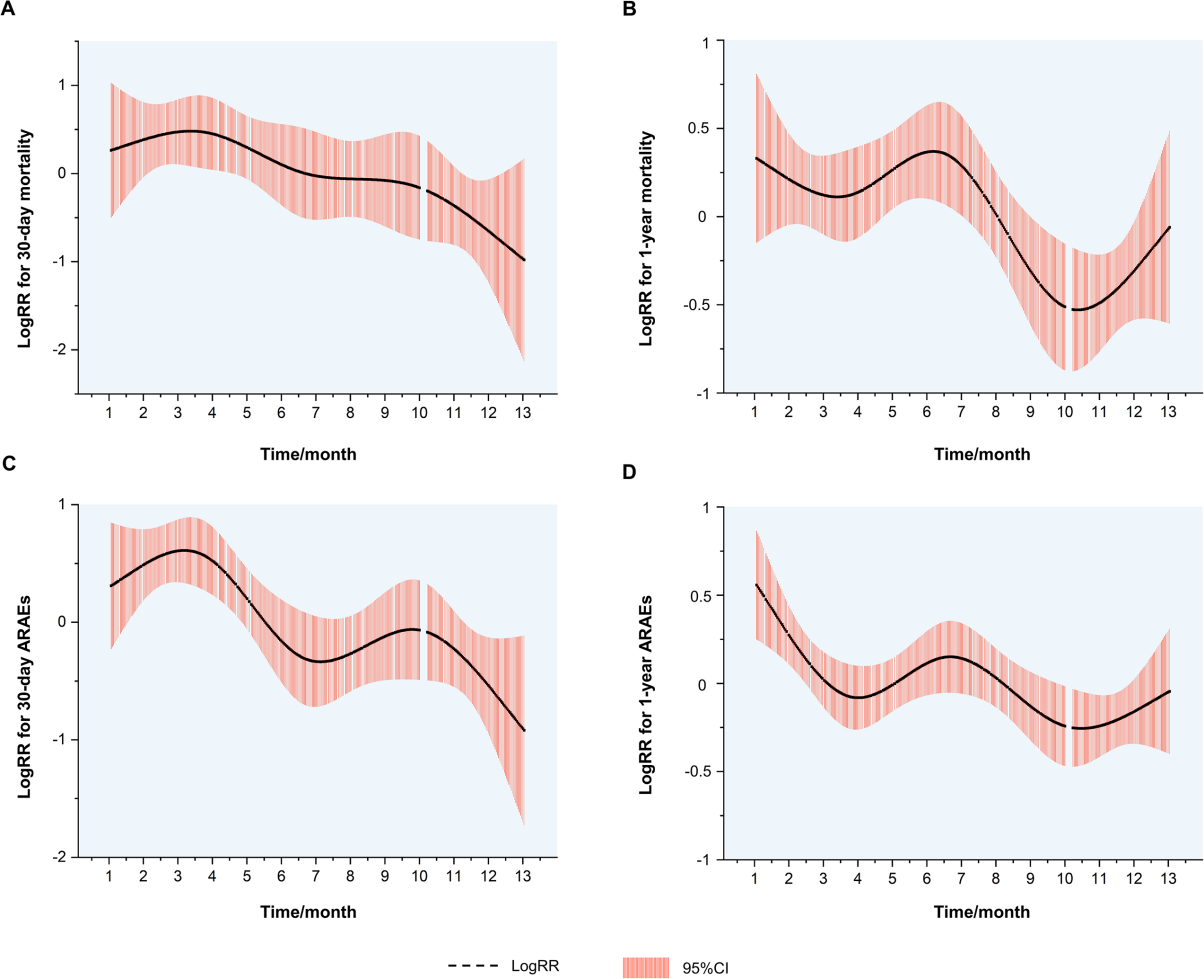
Kaplan–Meier curves. (**A**) Kaplan–Meier curves for the 30-day mortality. (**B**) Kaplan–Meier curves for the 1-year mortality. (**C**) Kaplan–Meier curves for the 30-day ARAEs. (**D**) Kaplan–Meier curves for the 1-year ARAEs. ARAEs, aortic-related adverse events.

The logRRs for ARAEs and all-cause mortality for different dates of TEVAR are demonstrated in Figure 3. It implied that the TEVAR intervention in autumn was positively correlated with a lower risk of 30-day ARAEs and 1-year mortality than in spring.

## Discussion

AD occurrence has been proven to have considerable diurnal and seasonal/monthly oscillations, similar to other cardiovascular diseases (14). Aside from the onset, the chronobiological patterns of acute AD may have a significant impact on patients’ prognoses. To our knowledge, this is the first retrospective clinical investigation to evaluate the relationship between the intervention seasons of TAVER and the outcome of TBAD patients. Our findings demonstrated that patients who received TEVAR in spring had higher risks of 30-day ARAEs and 1-year mortality than their counterparts in autumn (Figure 2). The impact of intervention seasons of TEVAR on 1-year all-cause mortality for TBAD patients remained significant after multivariate Cox analysis (Table 5), while the onset season did not influence the outcomes of TBAD patients (Supplementary Table S1).

Previous research has found that the onset of aortic dissection exhibited a peak in the winter and a bottom in the summer months (15, 16). The characteristic was most likely attributed to the temperature change. A new study offered convincing evidence that low ambient temperatures and temperature drops across nearby days were related to a higher risk of acute aortic dissection development (8). Our results show that the incidence of TBAD is higher in winter and early spring (Figure 1A), which was consistent with the previous study.

Despite this, spring and autumn are both considered moderate-temperature seasons, while autumn can be more dangerous due to the upcoming winter season. A previous study has shown that the onset of TAAD in autumn may be a factor that raises in-hospital mortality (10). Luo et al. found that patients admitted in autumn had an increased risk of in-hospital death following surgery for TAAD (11). However, previous scholarship failed to establish the contrast between the outcomes of TEVAR operated in spring and autumn. For the first time, our results show that TEVAR in spring is more dangerous than in autumn. The nonlinear fitted curves revealed that the risk of 30-day ARAEs reaches its peak in March and April (Figure 3C), while TEVAR in autumn has an evident advantage of lower 1-year mortality over spring (Figure 3B). This phenomenon contradicts common sense, which holds that a mild spring is suitable for surgery and favorable to wound healing, while a following freeze in autumn may worsen the situation.

The possible mechanisms are as follows: In contrast to previous studies (10, 11), patients with TAAD usually require open surgery, and a warmer climate after surgery is critical for massive invasive healing (11). Therefore, autumn is more dangerous for TAAD patients. However, for TEVAR, the minimally invasive technique is totally different. The effect of temperature on the aorta may be more important than that on the wound. When patients with aortic dissection and a frail aorta survive a cold winter, the accumulated stimulus and potential injury to the aorta reach a maximum. The temperature-lagging effect on cardiovascular diseases has been studied pretty consistently. The influence of heat was found to be rather instantaneous in most cases, but the effects of cold grew more prominent with greater time delays (17). The lagging effect of temperature on creatures has been found in a number of other fields (18, 19). As a result, there may be a high risk of aortic instability, making spring the riskiest season for TEVAR. This lagging effect can also be confirmed by the fact that autumn, rather than summer, is a seemingly safer season than spring.

The occurrence of the cumulative injury in winter may be mediated by two possibly synergistic processes including the sympathovagal balance and the hemorheological characteristics of circulating blood (20). A spike in sympathetic nerve activity accompanied by vagal nerve arrest led to an increase in heart rate and blood pressure in the cold winter (21). Simultaneously, the balance of thrombotic and thrombolytic factors gets altered, resulting in hypercoagulability (increased platelet aggregation, factor VII activity, and fibrinogen levels) and reduced fibrinolytic activity, all of which contribute to increased blood viscosity (22–24). On the contrary, all these effects may enhance the sympathetic tone and add force to the aorta. In addition, Szilágyi et al. found that the higher atmospheric pressure in winter may be another risk factor for increased mortality for acute aortic dissection patients (9). For TBAD patients, the accumulative injury puts great stress on the aorta, which is already undermined by the inherited and acquired factors. Thus, spring may be the weakest season for the aortic wall and a buffer for aortic remodeling. Furthermore, the sudden temperature swings and the possibility of a flu outbreak might also result in significant internal environmental changes and increased risks. Ashur et al. found that patients with acute TAAD who were hospitalized during the influenza season (spring) had higher in-hospital mortality (25).

TEVAR in the spring, on the other hand, is associated with a lower risk of 30-day endoleak than TEVAR in the autumn. While the mechanism is still unclear, one possibility is that endoleak is a unique ARAE that is more tied to the subsequent environment than to the season preceding TAVER. Endoleak may be more likely to occur when the temperature drops in autumn. The decreasing temperature may affect the thrombosis state and lead to endoleak (22, 26, 27). At the same time, the morphological properties of the stent graft can change with the introduction of cold current. All these may contribute to the endoleak after TEVAR.

In addition, socioeconomic factors may also influence the prognosis of patients. The patients we included mainly existed after 2008 (Supplementary Figure S1). As China's economy grew steadily, the income of the vast majority of patients and the national medical insurance policy might not change with seasonal variations. In addition, although earthquakes and other natural disasters occurred in western China in the spring of 2008 and 2012, the patients we followed up did not experience any of these. Furthermore, holidays and the Chinese Spring Festival could also have an influence. The Chinese Spring Festival is the largest traditional festival in China. It is also inevitable that sympathetic nerve excitation will increase during this period, increasing the possibility of cardiovascular-related deaths. Consequently, they may be confined to China.

Finally, climatic change may have a substantial influence on TBAD patients who have received TEVAR. Preoperative circumstances, rather than postoperative climate circumstances, may determine the stability of the aortic wall of TBAD patients. Preoperative warming is as crucial as postoperative warming for TBAD patients who receive TEVAR. In addition, the air pressure should also be closely monitored, as should protection against influenza infection. In previous investigations, early TEVAR treatment has been demonstrated to be superior to optimal medication therapy in the remodeling of the aorta for patients with acute, uncomplicated TBAD (4). However, preventive TEVAR for these patients in spring, according to our results, may add extra risk factors. Further research is required to validate the effectiveness of prophylactic TEVAR vs. medication treatment in winter and spring for acute, uncomplicated TBAD patients. Patients who must be treated in spring need more care and close follow-up.

### Limitation

This study has several limitations. (1) This study was a retrospective study. Data records for certain patients may be inaccurate, resulting in increased margins of error. (2) As a single-center study, generalization problems due to race and geographic restrictions, changes in socioeconomic situations induced by seasonal variations, such as festivals and vacations, and the risk of natural catastrophes should be interpreted cautiously to other regions or countries.

## Conclusion

Seasonal variations may have an impact on the outcome of TEVAR-treated TBAD patients. TEVAR administered in spring increases the risks of 30-day ARAEs and 1-year mortality than that in autumn.

## Data Availability

The raw data supporting the conclusions of this article will be made available by the authors, without undue reservation.
