# Supplementary material for: The intervention seasons of thoracic endovascular aortic repair impacted the outcomes for patients with type B aortic dissection
Source: Front Cardiovasc Med. 2023 Mar 21;10:1100075. doi: 10.3389/fcvm.2023.1100075 (PMC10071004; doi:10.3389/fcvm.2023.1100075)
Supplement: Supplementary file 1 [file Table1.docx]

**SUPPLEMENTAL TABLE. Cox Analyses of Mortality and ARAEs Stratified with Onset Seasons of TBAD**

|  | **Autumn** | **Spring** | | **Summer** | | **Winter** | |
| --- | --- | --- | --- | --- | --- | --- | --- |
|  |  | **HR (95% CI)** | **P-value** | **HR (95% CI)** | **P-value** | **HR (95% CI)** | **P-value** |
| 30-day mortality | 1 | 2.2 (0.4, 11.3) | 0.348 | 2.1 (0.4, 11.7) | 0.381 | 2.7 (0.6, 13.2) | 0.208 |
| 30-day ARAEs | 1 | 1.3 (0.5, 3.6) | 0.635 | 1.0 (0.3, 3.2) | 0.947 | 1.8 (0.7, 4.7) | 0.224 |
| 1-year mortality | 1 | 1.9 (0.8, 4.6) | 0.169 | 1.5 (0.6, 4.0) | 0.398 | 1.7 (0.7, 4.1) | 0.259 |
| 1-year ARAEs | 1 | 1.1 (0.6, 2.0) | 0.782 | 1.0 (0.6, 1.9) | 0.885 | 1.4 (0.8, 2.4) | 0.215 |

ARAEs = aortic-related adverse events; TBAD = type B aortic dissection; HR = hazard ratio; CI = confidence interval.
