# Supplementary figures and images for: The intervention seasons of thoracic endovascular aortic repair impacted the outcomes for patients with type B aortic dissection
Source: Front Cardiovasc Med. 2023 Mar 21;10:1100075. doi: 10.3389/fcvm.2023.1100075 (PMC10071004; doi:10.3389/fcvm.2023.1100075)

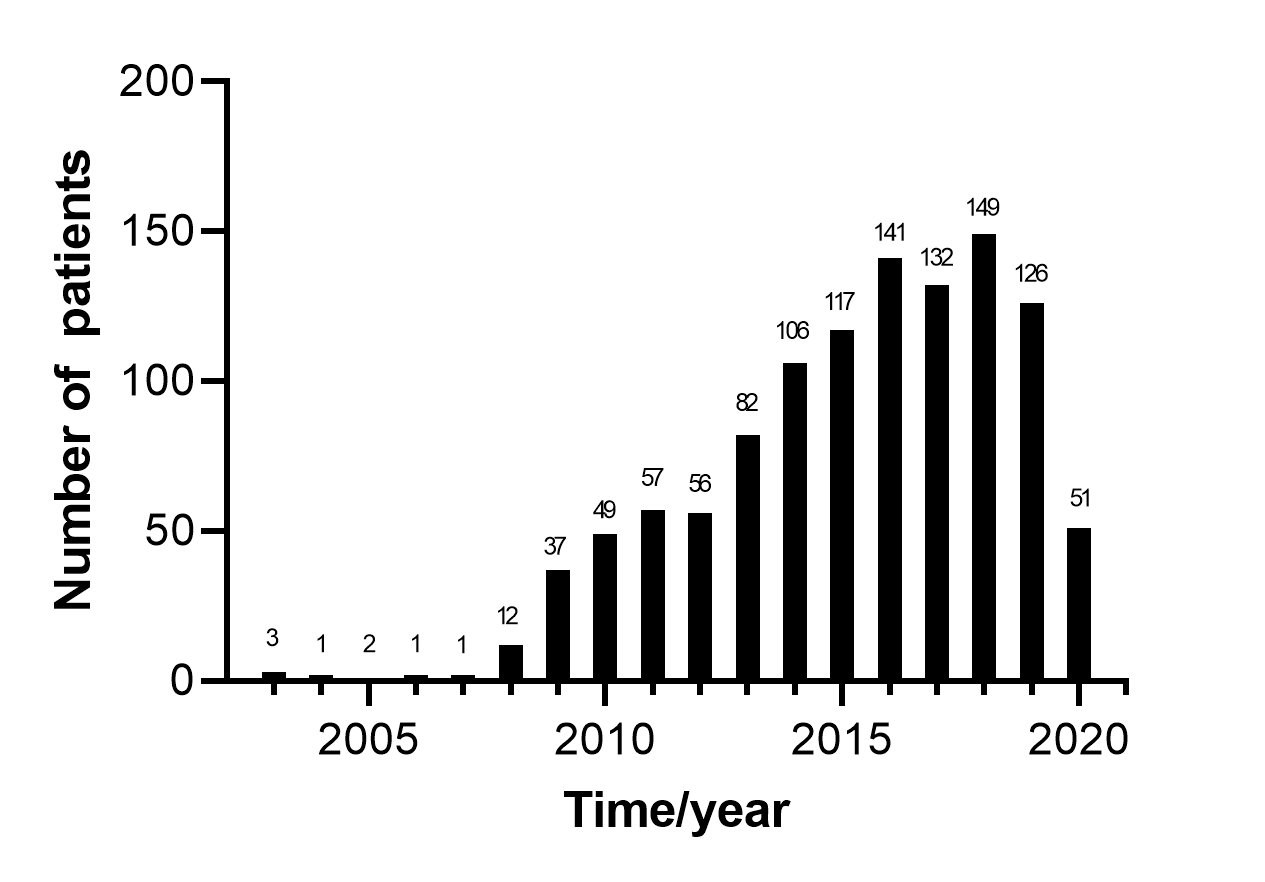

Supplement: Supplementary file 2 [file Image1.tif]

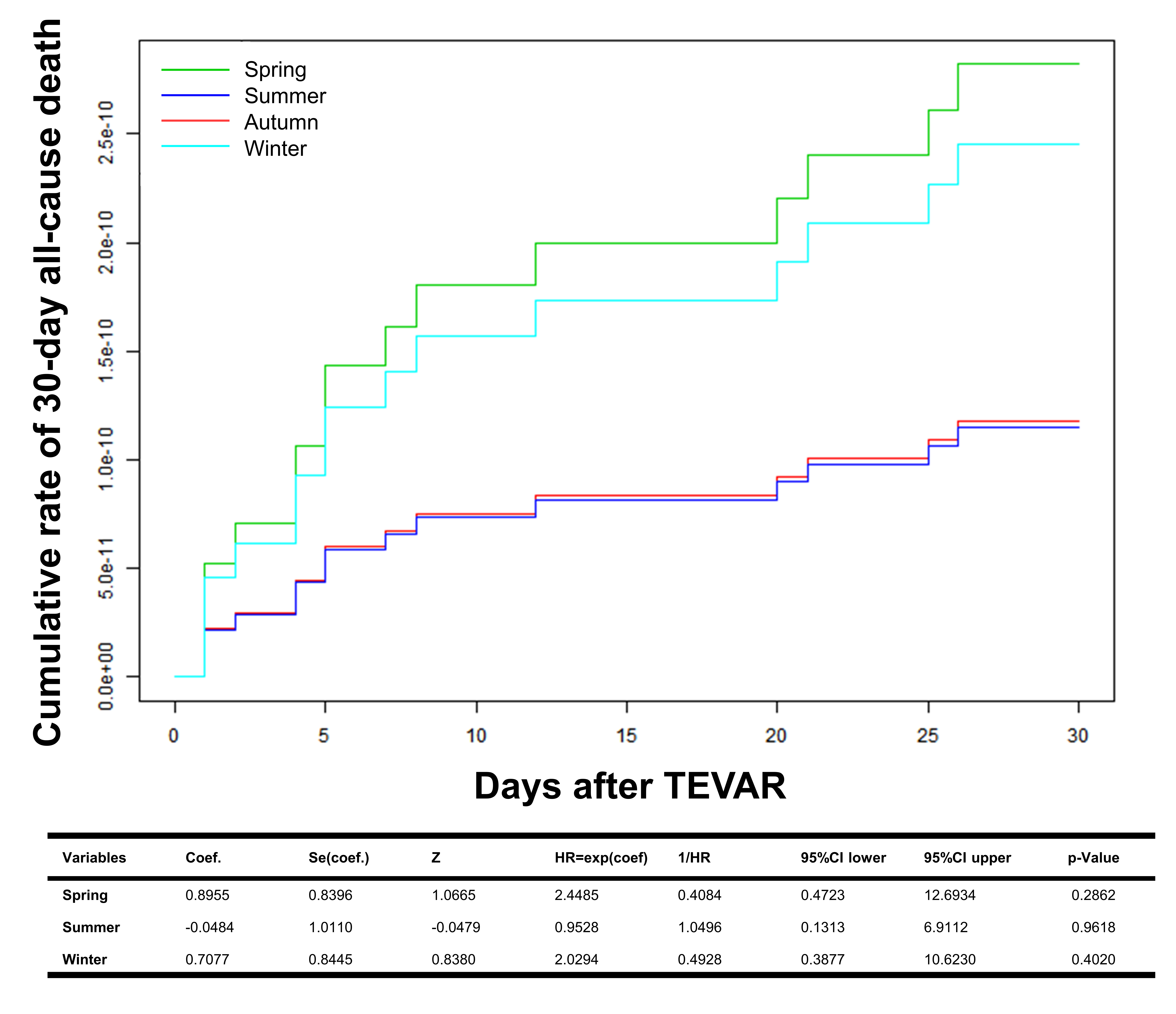

Supplement: Supplementary file 3 [file Image2.tif]

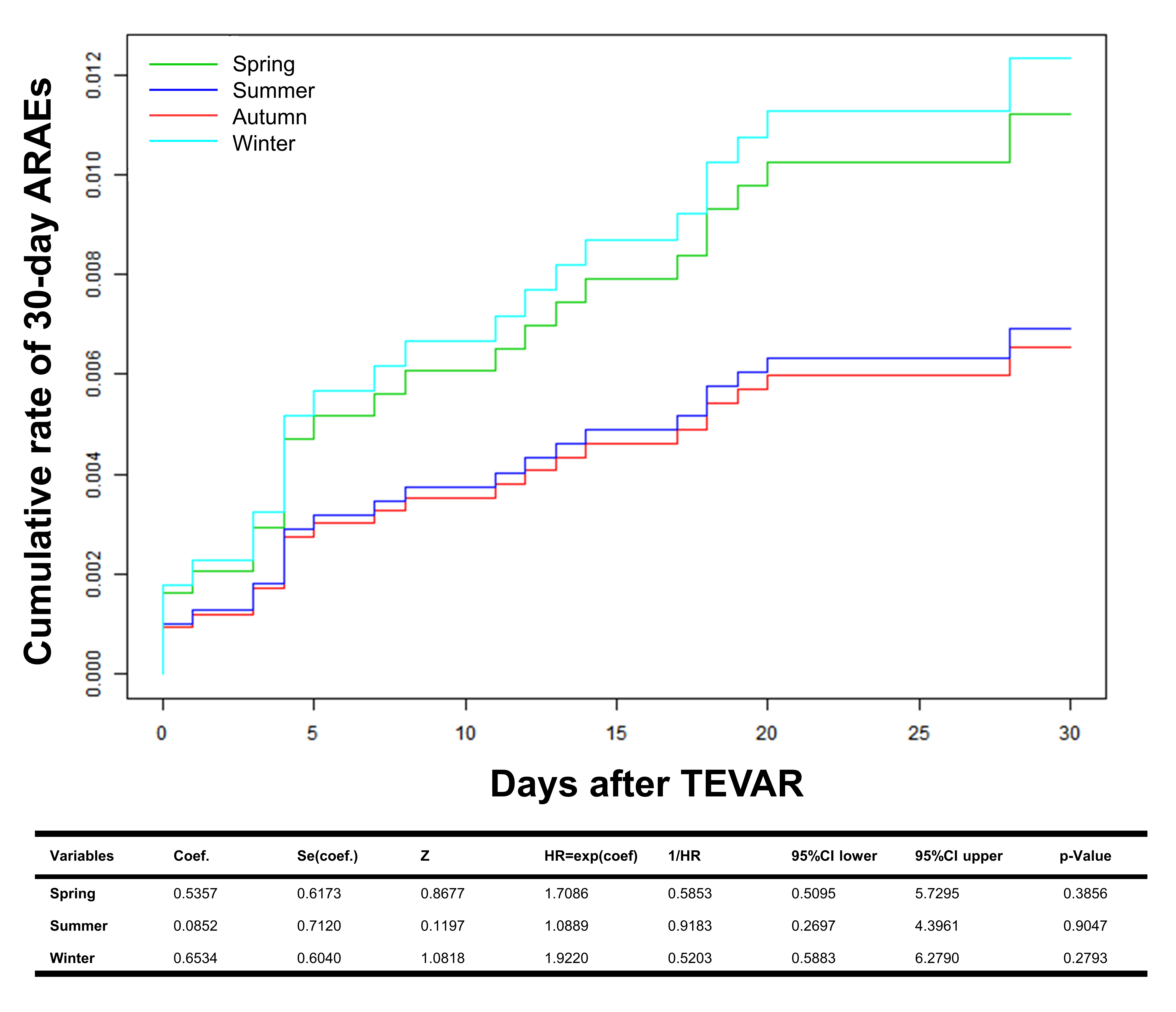

Supplement: Supplementary file 4 [file Image3.tif]

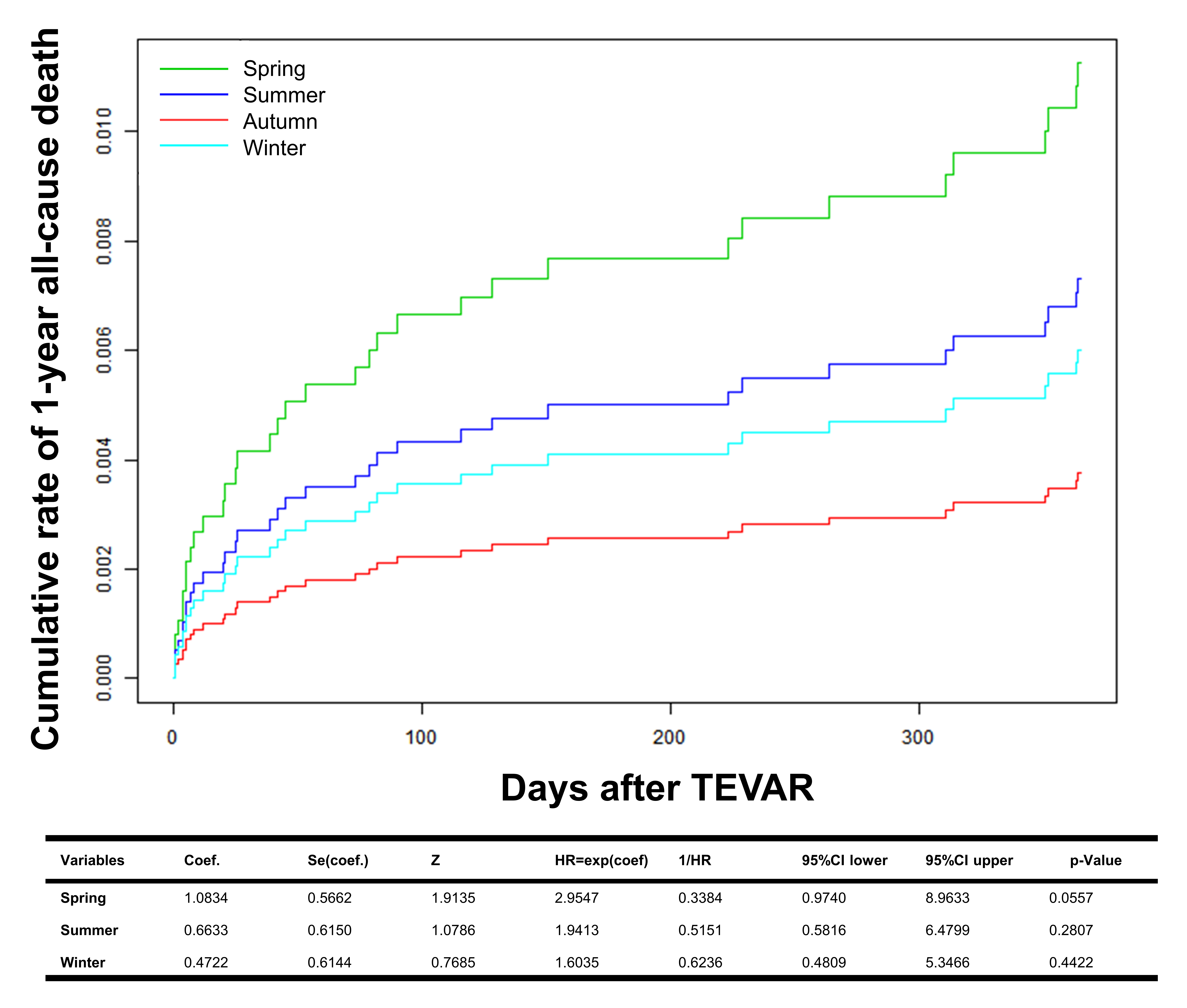

Supplement: Supplementary file 5 [file Image4.tif]

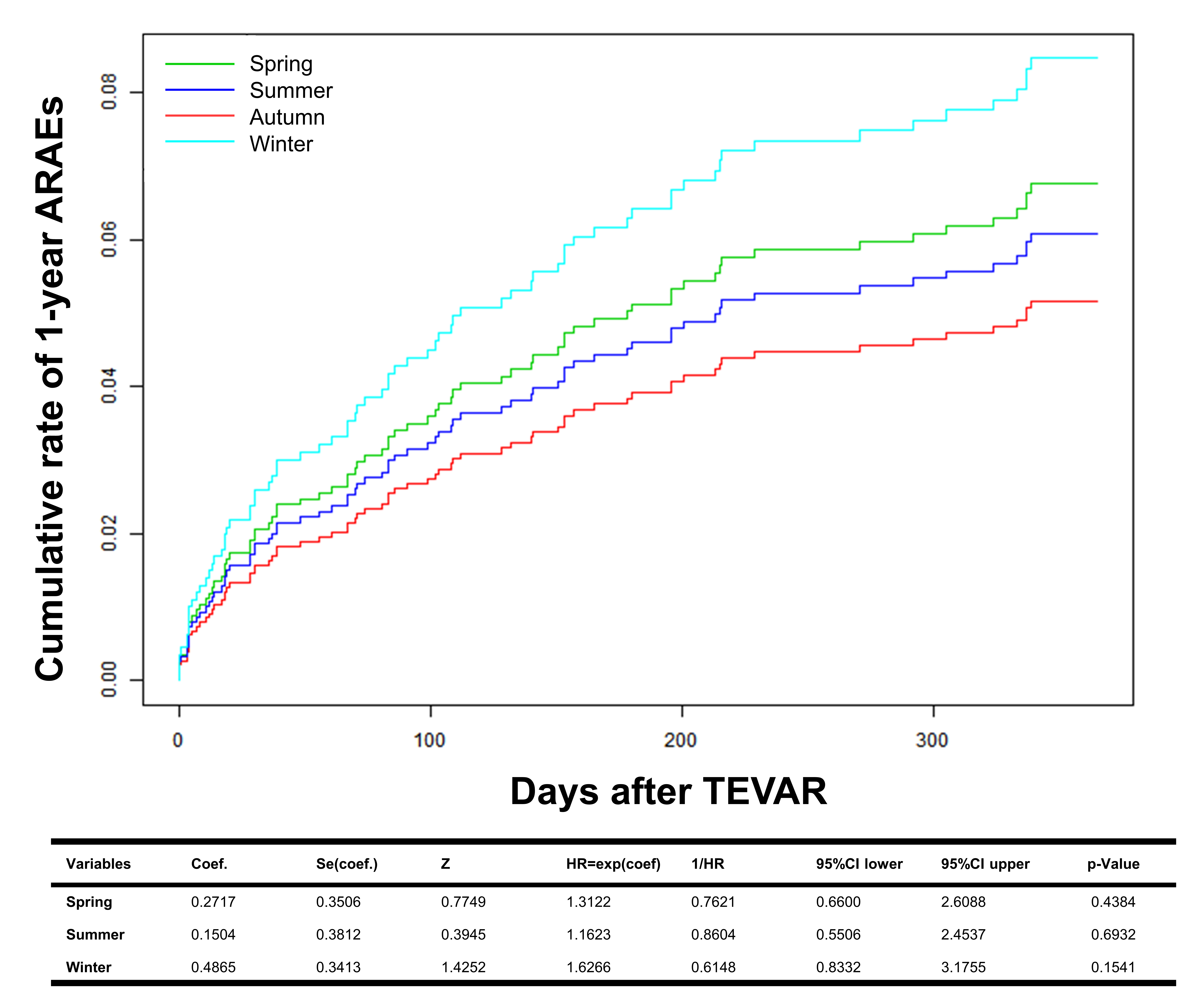

Supplement: Supplementary file 6 [file Image5.tif]
